# Multiple Introductions and Cross-Sector Transmission of *Salmonella* e*nterica* Serovar Infantis Carrying *bla*_CTX-M-65_ Gene, South Korea, 2022–2024

**DOI:** 10.3201/eid3210.251758

**Published:** 2026-10

**Authors:** Eunkyung Shin, Tae-Min La, Jaeil Yoo, Sungsuck Oh, Juhee Kim, Junyoung Kim, Ji-Yeon Hyeon

**Affiliations:** Division of Bacterial Diseases, Korea Disease Control and Prevention Agency, Cheongju, South Korea (E. Shin, J. Yoo, Junyoung Kim); College of Veterinary Medicine, Konkuk University, Seoul, South Korea (T.-M. La, J.-Y. Hyeon); Division of Emerging Infectious Diseases, Incheon Metropolitan City Institute of Public Health and Environment, Incheon, South Korea (S. Oh, Juhee Kim)

**Keywords:** Salmonella enterica, bacteria, antimicrobial resistance, food safety, multidrug-resistant *Salmonella* Infantis, *bla*_CTX-M-65,_ multiple international introductions, intersectoral genomic relatedness, South Korea

## Abstract

*Salmonella enterica* subspecies *enterica* serovar Infantis carrying the *bla*_CTX-M-65_ gene has been increasingly detected globally, including in South Korea, since 2022. We analyzed 65 *bla*_CTX-M-65_–positive *Salmonella* Infantis isolates from humans, poultry, and wastewater collected during 2022–2024 by using whole-genome sequencing and phylogenomic analysis. All isolates were multidrug resistant, commonly to aminoglycosides, β-lactams, quinolones, tetracyclines, amphenicols, and folate pathway inhibitors. Phylogenetic analysis revealed high genetic similarity among isolates from humans, poultry, and wastewater, which were intermingled within monophyletic clades with high bootstrap support, indicating close genomic relatedness across sectors. Bayesian phylogeographic reconstruction suggested multiple potential introductions during 2018–2022, including 1 lineage related to isolates from the United States (clade I) and others related to isolates from the United Kingdom (clade II). Those findings highlight the emergence and genomic diversity of *bla*_CTX-M-65_–positive *Salmonella* Infantis in South Korea and support the value of continued genomic surveillance across sectors.

*Salmonella enterica* subspecies *enterica* serovar Infantis has emerged as a leading serovar of human salmonellosis (*1*) and is listed among the top 15 *Salmonella* serovars globally (*2*). Poultry and poultry-derived products are major reservoirs for this serovar, which has shown a steady global increase over the past decade, driven by its adaptation to poultry production systems and persistence throughout the food chain (*3*). Concurrently, multidrug-resistant (MDR) and extended-spectrum β-lactamase (ESBL)–producing *Salmonella* Infantis strains have been increasingly reported, posing a growing public health concern (*4*). Those resistant clones have emerged through acquisition of large plasmids carrying multiple resistance and virulence determinants. One of the earliest reports, from Israel in 2008, described an ≈280-kb megaplasmid, termed a plasmid of emerging *Salmonella* Infantis (pESI) (*5*). That plasmid harbored multiple resistance genes, including genes conferring resistance to tetracycline, trimethoprim, sulfamethoxazole, antiseptics, and heavy metals, as well as virulence factors, such as yersiniabactin (*ybt* operon) and fimbrial operons (*fea* and *ipf*) that enhance survival, adaptation, and colonization under antimicrobial and environmental stress, strengthening bacterial fitness (*5*). pESI-like plasmids have rapidly disseminated worldwide, evolving to carry ESBL genes such as *bla*_CTX-M-1_ and *bla*_CTX-M-65_. *Salmonella* Infantis carrying *bla*_CTX-M-65_ was reported from fecal samples collected from children in Peru during 2012–2013 (*6*); subsequently spread across humans, food animals, and retail meat in the United States (*7*); and later was detected from human and food animal samples in Chile (*8*). Those reports indicated that *bla*_CTX-M-65_–positive *Salmonella* Infantis had emerged in humans, food animals, and food products, highlighting its relevance for One Health surveillance.

In South Korea, isolates collected through the national gastrointestinal infection surveillance networks (EnterNet Korea) have shown an increase in *Salmonella* Infantis since 2022 (*9*). More recently, *bla*_CTX-M-65_–positive *Salmonella* Infantis has been increasingly identified in the poultry sector (*10*). However, genomic characterization from human sources remains limited, with only 1 human isolate reported to date (*11*). Thus, although recent studies have documented the emergence of this lineage in South Korea, integrated genomic data spanning human, poultry, and environmental sources remain scarce. That gap has limited understanding of the origins, phylogenomic relationships, and public health implications of *bla*_CTX-M-65_–positive *Salmonella* Infantis in South Korea. To address that gap, we conducted whole-genome sequencing (WGS) and phylogenomic analysis of *bla*_CTX-M-65_–positive *Salmonella* Infantis isolates from humans, poultry, and wastewater in South Korea and compared those isolates with global genomes to investigate genetic relationships and phylogenomic context of this bacterium.

## Materials and Methods

### Bacterial Isolates

We analyzed a total of 65 *bla*_CTX-M-65_–positive *Salmonella* Infantis isolates collected during 2022–2024, including 45 from human patients, 16 from poultry, and 4 from wastewater. Human isolates were collected through national surveillance programs (EnterNet and PulseNet Korea) maintained by the Korea Disease Control and Prevention Agency. During 2022–2024, a total of 2,817 human *Salmonella* isolates were collected, of which 258 were identified as *Salmonella* Infantis. From those, we included all 45 ESBL-producing *Salmonella* Infantis isolates in this study.

Wastewater isolates were obtained from influent samples collected at 2 public wastewater treatment facilities (Songdo 2 and Namhang) in Incheon through a wastewater-based infectious disease surveillance program. Through that surveillance program, 4 ESBL-producing *S*. Infantis isolates were identified during the study period; we included all 4 in this study.

We obtained poultry isolates collected from chicken fecal samples from poultry farms through the foodborne pathogen tracking and monitoring program. During 2023, a total of 40 poultry-derived *Salmonella* isolates were collected from 32 poultry farms, of which 20 *Salmonella* Infantis isolates were recovered from 17 farms. Among those, 16 ESBL-producing *Salmonella* Infantis isolates from 16 farms were collected, and we included all 16 in this study (Appendix Table 1).

We performed species identification by using the VITEK-II automated system (bioMérieux, https://www.biomerieux.com) and conducted serotyping according to the Kauffmann–White scheme with specific antiserum (BD Biosciences, https://www.bdbiosciences.com). We performed antimicrobial susceptibility testing by broth microdilution using customized Sensititer KRCDC2F plates (TREK Diagnostic Systems, https://www.trekds.com), according to Clinical and Laboratory Standards Institute guidelines (*12*).

### WGS

We used the DNeasy Blood and Tissue Kit (QIAGEN, https://www.qiagen.com) to extract genomic DNA. We performed WGS on a MiSeq platform (Illumina Inc., https://www.illumina.com) by using the MiSeq Reagent Kit v2 (Illumina) at 500 cycles for 2 × 250-nt reads. We used Trimmomatic version 0.36 (*13*) to process reads, then SPAdes version 3.15.5 (*14*) to assemble trimmed raw reads. We confirmed serovar prediction by using sistr_cmd version 1.1.2 (https://github.com/phac-nml/sistr_cmd). We used ABRicate version 1.0.1 (https://github.com/tseemann/abricate) with the ResFinder database (*15*) to identify antimicrobial resistance (AMR) genes and with a custom database comprising 283 genes from the reference strain 119944 (GenBank accession no. CP047882) to identify pESI-like megaplasmid markers. For additional plasmid characterization, we randomly selected 1 human isolate (23SAL-3252) and 1 poultry isolate (SAL-AN014) from clade II for long-read sequencing. We used the Ligation Sequencing Kit to prepare libraries and performed sequencing on a MinION platform with R10.4.1 flow cells using MinKNOW software (all Oxford Nanopore Technologies, https://nanoporetech.com) with default settings. We used Autocycler version 0.6.1 (*16*) to assemble nanopore reads, then used Unicycler_polish (*17*) to polish with the corresponding Illumina reads under default parameters. For clade I, we assessed plasmid context by using previously reported complete genome sequences from retail chicken meat in South Korea (*18*). In addition, to illustrate broader structural variation and conserved AMR gene organization, we used Proksee (https://proksee.ca) to compare previously defined pESI-like plasmid variants from South Korea (*18*) with the clade II plasmid sequences from this study.

### Phylogenetic Analysis

To investigate the genetic relationships of our 65 isolates, we constructed 2 whole-genome single-nucleotide polymorphism (wgSNP) phylogenetic trees. One tree compared our 65 isolates with 37 *bla*_CTX-M-65_–positive *Salmonella* Infantis isolates from South Korea; the other compared our isolates with a total of 2,305 *bla*_CTX-M-65_–positive *Salmonella* Infantis isolates, including the 37 from South Korea used in the first tree and 2,268 global genomes from EnteroBase (https://enterobase.warwick.ac.uk).

To perform the phylogenetic analysis of South Korea isolates, we downloaded 52 additional *Salmonella* Infantis genomes collected from South Korea from GenBank in October 2024 and evaluated genomes by using sistr_cmd version 1.1.2 (Appendix Table 2). After quality filtering, we retained 16 genomes harboring *bla*_CTX-M-65_ and pESI-like plasmids and used those for further analysis. In addition, the College of Veterinary Medicine, Konkuk University, Seoul, South Korea, provided 21 genomes of *bla*_CTX-M-65_–positive *Salmonella* Infantis collected from poultry isolates in 2023. For the global phylogenetic analysis, we retrieved 2,723 *Salmonella* Infantis genomes carrying *bla*_CTX-M-65_ from EnteroBase in October 2024 by using the search terms Infantis (serovar) and *bla*_CTX-M-65_ (AMR analysis). After quality evaluation with sistr_cmd version 1.1.2 and metadata filtering, we included a total of 2,268 strains for further analysis.

We identified wgSNPs by using Snippy version 4.6.0 (https://github.com/tseemann/snippy) and used *Salmonella* Infantis N55391 (GenBank accession no. CP016410) as the reference. We used PHASTEST (*19*) to identify prophages and the Nucmer tools from the Mummer package version 3.23 (*20*) to identify repetitive regions within the reference genome. We excluded single-nucleotide polymorphisms (SNPs) located in prophage and in repetitive regions. We used Gubbins version 3.3.1 (*21*) to identify and subsequently mask recombination regions from the wgSNP alignment before phylogenetic analysis. We used IQ-TREE with a TVMe model selected by ModelFinder (*22*) and 1,000 bootstrap replicates to infer the maximum-likelihood tree for the South Korea dataset and used FastTree (*23*) to infer the maximum-likelihood tree for the global dataset.

### Bayesian Phylogenetic Analysis

To reconstruct the evolutionary history, we identified a subtree consisting of 101 strains from South Korea and 116 strains from 12 other countries on the basis of a FastTree support value of >0.9. Of those, we included 97 domestic and 79 international strains (Appendix Table 3) with available FASTQ data in the analysis, for a total of 176 strains. We downloaded raw reads from the National Center for Biotechnology Information Sequence Read Archive (https://ncbi.nlm.nih.gov/sra) by using fasterq-dump (https://github.com/ncbi/sra-tools), then quality-trimmed and processed reads for SNP calling as described.

We used TempEst version 1.5.3 (*24*) to assess the temporal signal (*R*^2^ >0.8561). We performed Bayesian phylogenetic analysis by using BEAST version 1.10.4 (*25*) with a TVMe substitution model selected by using ModelTest-NG (*26*). We used path and stepping-stone sampling (*27*) to evaluate marginal likelihood of 2 clock models, constant and lognormal relaxed; and 3 tree models, constant population size, exponential growth, and Gaussian Markov random field Bayesian Skyride model. The lognormal relaxed clock model with Gaussian Markov random field Bayesian Skyride model yielded the highest Bayes factor (BF), indicating the best fit.

For discrete trait phylogeographic analysis, we reconstructed ancestral locations and estimated asymmetric exchanges between regions by using a nonreversible continuous-time Markov chain model. We excluded the United States and Italy because those countries were each represented by 1 sequence. To identify well-supported transitions between discrete states, we used Bayesian stochastic search variable selection and Bayes factor (BF) testing, as implemented in SPREAD3 software version 0.9.6 (*28*). We considered transitions with posterior probability >0.5 and BF >3 significant (*28*). We used stochastic mapping (*29*) to estimate transition rate and count (Markov jumps). We ran 3 independent chains of 100 million generations in parallel with sampling every 10,000 generations. We evaluated the results in Tracer (http://beast.community/tracer) to ensure that effective sample sizes were >200 and to ensure convergence between all 3 runs. We used LogCombiner version 1.10.4 (https://beast.community/logcombiner) to combine the log and tree files from 3 runs, with 10% burn-in. We used TreeAnnotator version 1.10.4 (https://beast.community/treeannotator) to generate a maximum clade credibility tree with 10% burn-in and common ancestor node heights. We annotated the maximum clade credibility tree by using FigTree version 1.4.4 (https://tree.bio.ed.ac.uk/software/figtree).

To evaluate the potential effects of unequal sampling across discrete geographic states, we used a generalized linear model as an extension of phylogeographic inference in BEAST version 1.10.4 (*25*). Coefficients quantified the effect size, and we used Tracer version 1.7.1 to analyze indicators that determined predictor inclusion. We used the number of sequences assigned to each discrete state as predictors to inform transition rate estimates.

## Results

### Phenotypic and Genotypic AMR

We assessed antimicrobial susceptibility profiles for the 65 *bla*_CTX-M-65_–positive *Salmonella* Infantis isolates from South Korea (Appendix Table 4). All isolates were resistant to ampicillin, cefotaxime, ceftriaxone, and tetracycline but remained susceptible to azithromycin and amikacin. We observed high resistance rates for nalidixic acid (98.5%; n = 64), chloramphenicol (93.8%; n = 61), gentamicin (81.5%; n = 53), and trimethoprim/sulfamethoxazole (80.0%; n = 52). All isolates were resistant to >3 antimicrobial classes and thus classified as MDR (Table). The predominant resistance profile, observed in 43 (66.2%) isolates, included resistance to aminoglycosides, β-lactams, quinolones, tetracyclines, amphenicols, and folate pathway inhibitors. Of note, 1 human isolate displayed resistance to 7 antimicrobial classes.

**Table:** Antimicrobial resistance patterns of 65 isolates from a study of multiple introductions and cross-sector transmission of *Salmonella* e*nterica* serovar Infantis carrying *bla*_CTX-M-65_ gene, South Korea, 2022–2024

Antimicrobial resistance pattern	No. (%) isolates
β-lactams–quinolones–tetracyclines	2 (3.1)
β-lactams–tetracyclines–amphenicols	1 (1.5)
β-lactams–quinolones-tetracyclines–amphenicols	2 (3.1)
Aminoglycosides–β-lactams-quinolones–tetracyclines	1 (1.5)
β-lactams–quinolones–tetracyclines–amphenicols–folate pathway inhibitors	7 (10.8)
Aminoglycosides–β-lactams–quinolones–tetracyclines–folate pathway inhibitors	1 (1.5)
Aminoglycosides–β-lactams–quinolones–tetracyclines–amphenicols	7 (10.8)
Aminoglycosides–β-lactams–quinolones–tetracyclines–amphenicols–folate pathway inhibitors	43 (66.2)
Aminoglycosides–β-lactams–quinolones–polymyxins–tetracyclines–amphenicols–folate pathway inhibitors	1 (1.5)

We evaluated AMR gene profiles of 102 *bla*_CTX-M-65_–positive *Salmonella* Infantis isolates from South Korea, including the 65 isolates from this study (Figure 1). All isolates harbored >3 distinct aminoglycoside resistance genes. The most prevalent genes were *aph*(4)-Ia (100%), *aac*(3)-Iva (100%), *aac*(6′)-Iaa (99%), *ant*(3″)-Ia (98%), and *aph*(3′)-Ia (68%). All the isolates carried *tet*(*A*), whereas we detected *sul1* in 98%, *floR* in 91%, and *dfrA14* in 71% of the isolates. In addition, we identified *bla*_TEM-30_ in 1 human isolate.

**Figure 1:**
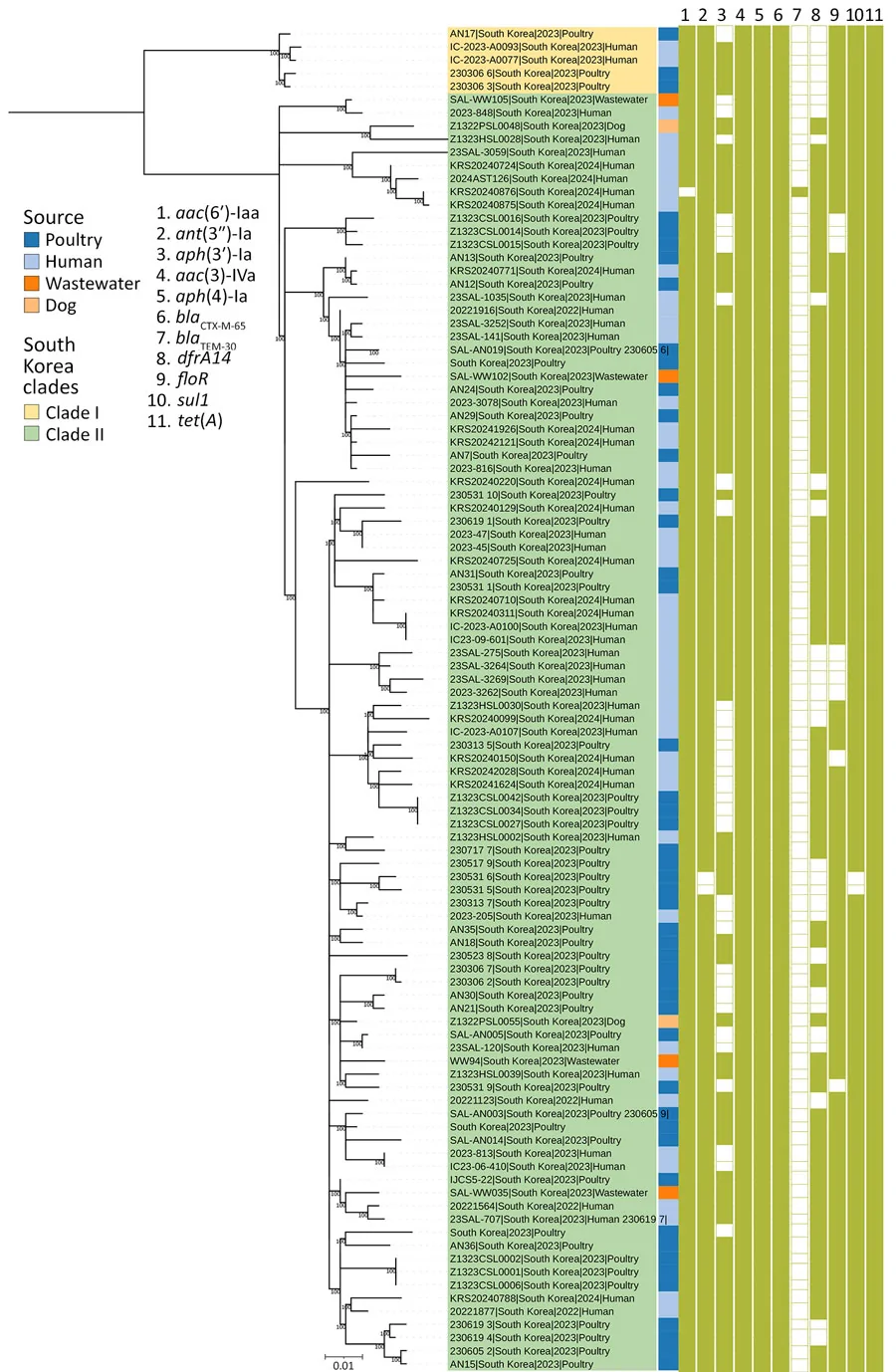
Maximum-likelihood phylogenetic tree for 102 isolates from study of multiple introductions and cross-sector transmission of *Salmonella* e*nterica* serovar Infantis carrying *bla*_CTX-M-65_ gene, South Korea, 2022–2024. South Korea clades I and II were defined as the 2 major groups observed in the midpoint-rooted phylogeny. Antimicrobial resistance genes are annotated. Numbers at nodes indicate bootstrap support values >90%. Pairwise single-nucleotide polymorphism distances within and between clades are available in Appendix Figure 1. Scale bar indicates nucleotide substitutions per site.

### Phylogenetic Analysis of *bla*_CTX-M-65_–Positive *Salmonella* Infantis Isolates

The final alignment of the 2 wgSNP phylogenetic trees included 687 SNP sites in the South Korea dataset and 12,616 SNP sites in the global dataset (data not shown). The South Korea genomes formed 2 distinct clades (clade I and clade II) (Figure 1). Clade I comprised 5 isolates, including 2 from humans and 3 from poultry, all collected in 2023, with a median of 5 (range 3–7) SNPs difference (Appendix Figure 1). Clade II contained 97 isolates from human, poultry, and wastewater sources interspersed throughout the clade, with a median of 28 (range 0–60) SNPs difference (Appendix Figure 1). The median pairwise distance between clades I and II was 69 (range 60–81) SNPs (Appendix Figure 1).

In the global phylogeny, all South Korea isolates were separated into 2 major clades (Figure 2), consistent with the national-level analysis. Clade II isolates from South Korea clustered with 116 isolates from 12 countries, whereas clade I isolates clustered with 15 isolates from the United States and 2 others from South Korea. Considering the broad international distribution of clade II isolates, we conducted a phylogeographic analysis to determine their potential geographic origins and transmission routes.

**Figure 2:**
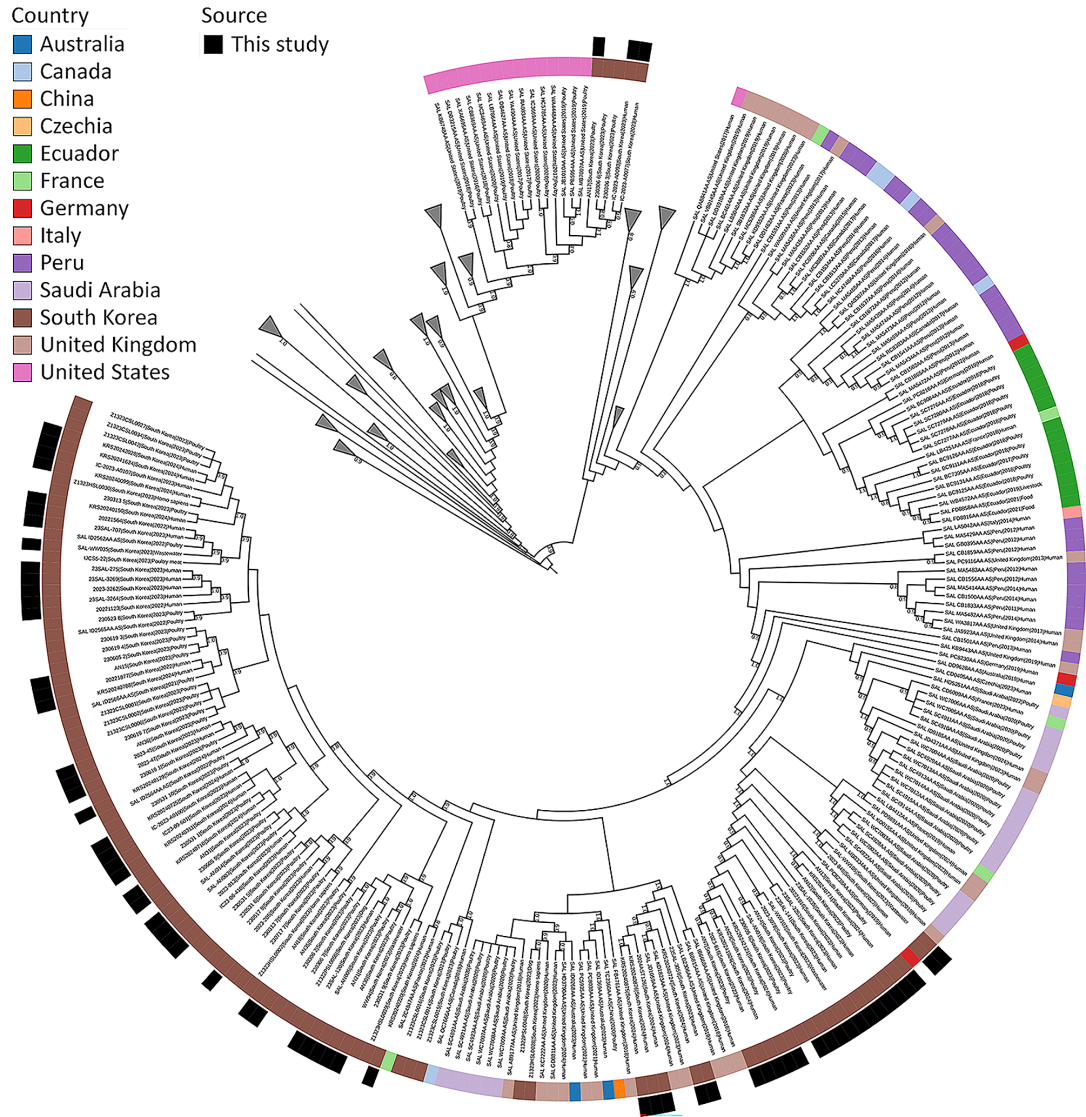
Global phylogenetic placement of isolates from study of multiple introductions and cross-sector transmission of *Salmonella* e*nterica* serovar Infantis carrying *bla*_CTX-M-65_ gene, South Korea, 2022–2024. The cladogram was inferred from the global whole-genome single-nucleotide polymorphism dataset, and branches without South Korea isolates were collapsed for clarity. The inner ring denotes country of origin, and the outer ring denotes isolates sequenced in this study. Numbers at nodes indicate FastTree (*23*) support values >0.9.

All South Korea and related global isolates carried 256–283 of the 283 genes in the reference genome and harbored all 6 hallmark loci (i.e., *ardA*, *pilL*, *sogS*, *trbA*, *ipf*, and *ipr2*) (*30*), confirming the presence of a pESI-like plasmid (data not shown). In addition, long-read sequencing and assembly of 2 randomly selected clade II isolates, 1 human and 1 poultry isolate, confirmed that *bla*_CTX-M-65_ was located on pESI-like megaplasmids. For clade I, previously reported complete genome sequences of MDR *Salmonella* Infantis carrying *bla*_CTX-M-65_ from retail chicken meat in South Korea showed similar pESI-like plasmid structures and resistance gene organization, despite structural variation among the plasmid variants (Appendix Figure 3).

### Phylogeographic Analysis of *bla*_CTX-M-65_–Positive *Salmonella* Infantis Isolates

Bayesian phylogenetic reconstruction of the global clade revealed 6 South Korea subclades (A–F), each strongly supported by a posterior probability of 1 (Figure 3; Appendix Figure 2). The estimated times to most recent common ancestor were 2019.29 (highest posterior density [HPD] 2018.58–2020.15) for clade A (n = 67), 2021.59 (HPD 2020.67–2022.51) for clade B (n = 3), 2020.07 (HPD 2019.24–2020.98) for clade C (n = 17), 2022.02 (HPD 2021.03–2022.81) for clade D (n = 2), 2020.07 (HPD 2019.24–2020.98) for clade E (n = 7, including 2 from the United Kingdom), and 2020.06 (HPD 2019.62–2021.66) for clade F (n = 2).

**Figure 3:**
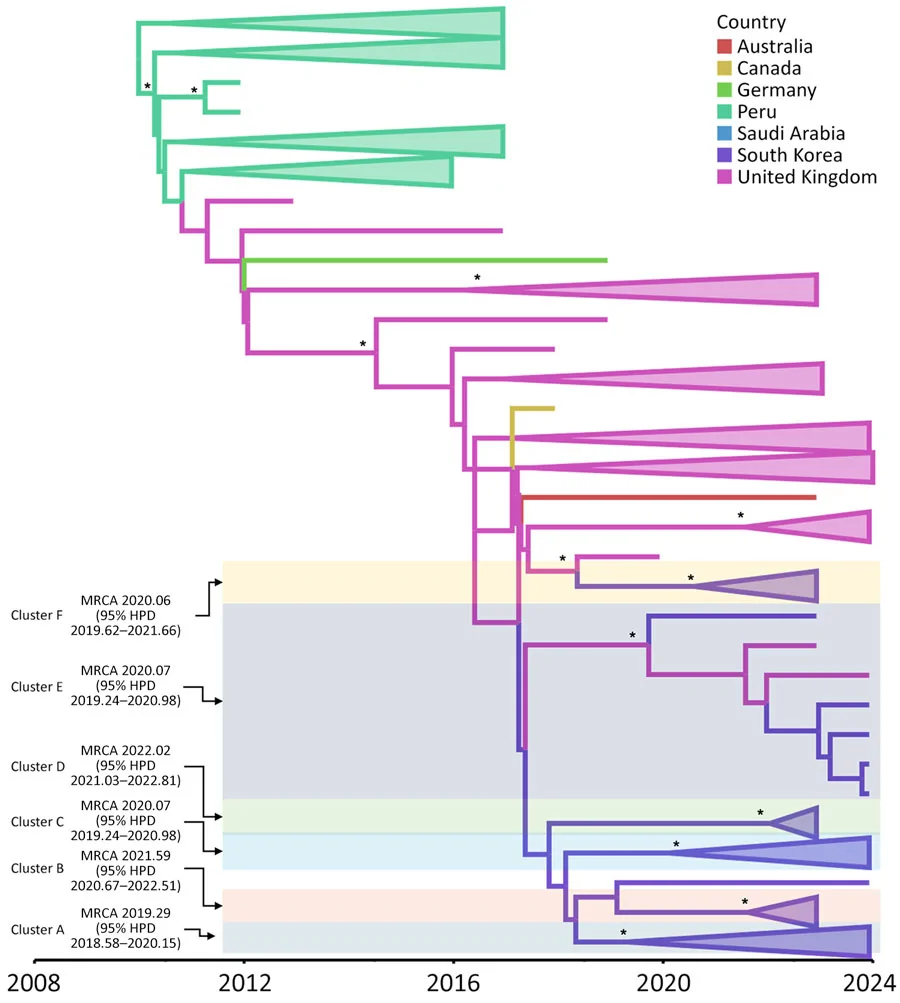
Bayesian phylogenetic tree from study of multiple introductions and cross-sector transmission of *Salmonella* e*nterica* serovar Infantis carrying *bla*_CTX-M-65_ gene, South Korea, 2022–2024. The geographic origin was treated as a discrete trait in the analysis. Branches are colored according to geographic origin. Branches without South Korea isolates were collapsed for clarity. South Korea clusters supported by a posterior probability of 1 were also collapsed to improve readability. Asterisks indicate clusters with posterior probability of 1. South Korea clusters are highlighted in color, with the inferred median MRCA age for South Korea isolates along with the 95% HPD interval. The full phylogeny is available in Appendix Figure 2. HPD, highest posterior density; MRCA, most recent common ancestor.

Bayesian phylogeographic analysis suggested that South Korea lineages were most closely related to isolates from the United Kingdom, with strong statistical support for this transition pattern (BF 983.911; posterior probability 0.994; transition rate 3.853 [95% HPD 2–5]). The generalized linear model analysis indicated minimal sampling bias because effects of source and sink sample sizes were negligible (source indicator 3.185 × 10^–3^; source coefficient −1.77 × 10^–3^; sink indicator 1.148 × 10^–3^; and sink coefficient −7.949 × 10^–3^).

## Discussion

This study provides a comprehensive genomic analysis of *bla*_CTX-M-65_–positive *Salmonella* Infantis in South Korea, revealing its recent emergence, genetic characteristics, and potential transmission routes. Our findings indicate close phylogenetic relatedness among isolates from humans, poultry, and wastewater, consistent with intersectoral circulation of closely related lineages.

Among the 65 *bla*_CTX-M-65_–positive *Salmonella* Infantis isolates, all exhibited resistance to multiple antimicrobial classes, including β-lactams, quinolones, tetracyclines, amphenicols, and folate pathway inhibitors. National surveillance conducted during 2016–2017 reported no MDR *Salmonella* Infantis isolates (*31*). Likewise, a regional surveillance study conducted in Seoul during 2020–2022 identified no MDR *Salmonella* Infantis isolates (*32*). Together, those findings highlight the recent emergence and dissemination of MDR *Salmonella* Infantis in South Korea, consistent with detection of ESBL-producing *Salmonella* Infantis carrying pESI-like plasmids in poultry in South Korea in 2021 and 2022 (*10*). The pESI-like plasmid might enhance stress tolerance and persistence, conferring an advantage to this clone over non-MDR strains.

The *bla*_CTX-M-65_–positive *Salmonella* Infantis isolates in this study carried a conserved resistance gene repertoire, with frequent additional determinants for aminoglycosides, tetracyclines, phenicols, sulfonamides, and trimethoprim. Similar multidrug-resistance repertoires have been reported in *Salmonella* Infantis lineages from multiple geographic settings, including lineages associated with pESI-like plasmids (*33*). Such a stable yet adaptable genetic background likely supports the long-term persistence and ecologic success of *Salmonella* Infantis across hosts.

The close genetic relatedness of wastewater isolates to human and poultry isolates suggests that closely related strains were in all sectors. However, the limited number of wastewater isolates warrants cautious interpretation regarding the role of wastewater as an environmental reservoir. Consistent with recent evidence of global cross-sector clustering (*4*,*34*), those findings underscore the interconnectedness of human, animal, and environmental reservoirs. Integrating genomic characterization into surveillance frameworks could improve tracking of *bla*_CTX-M-65_–positive *Salmonella* Infantis across sectors.

Globally, emergent *Salmonella* Infantis carrying pESI-like plasmids has been shown to form geographically structured lineages, with different subpopulations associated with distinct ESBL-gene variants, including *bla*_CTX-M-1_ and *bla*_CTX-M-65_ (*35*). All isolates from South Korea in this study belonged to ST32, supporting the emergence of a *bla*_CTX-M-65_–positive ST32 lineage in South Korea. Distinct pESI-like multidrug-resistant lineages, including ST2283, have been reported in other geographic settings, supporting the broader international diversity of pESI-like *Salmonella* Infantis (*4*). Our phylogeographic analysis suggested more recent introduction of *bla*_CTX-M-65_–positive *Salmonella* Infantis into South Korea during 2018–2022, comprising 4 potential introduction events: 1 from the United States (clade I) and 3 from the United Kingdom (clade II, supported by Bayesian discrete trait analysis with a transition rate of 3.853). That finding raises the possibility that importation of grandparent and parent stock poultry from those countries could represent an introduction route, consistent with previous reports linking the poultry trade to the spread of *bla*_CTX-M-65_–positive *Salmonella* in Taiwan and North America (*36*,*37*). Such introductions are consistent with a possible role for global trade in the transboundary spread of high-risk MDR clones (*38*), and parallel the recent emergence of a distinct *Salmonella* Enteritidis lineage in South Korea (*39*), which also might have been influenced by international transmission. During 2015–2023, South Korea imported ≈165,000 live chickens annually from the United States (≈$6.81 million US) and ≈93,000 from the United Kingdom (≈$3.26 million US) (*40*). Those findings support the integration of genomic characterization into existing surveillance frameworks in South Korea, including EnterNet Korea, PulseNet Korea, the wastewater-based infectious disease surveillance program, and poultry-associated foodborne pathogen monitoring programs. Such integration could enhance the early detection of emerging multidrug-resistant *Salmonella* Infantis lineages, strengthen source-attributed public health risk assessment, and support risk-based monitoring in poultry production and import settings.

The first limitation of our study is that direct epidemiologic links between human and poultry cases were not available; therefore, genomic clustering across sectors should not be interpreted as evidence of direct transmission or transmission directionality. Second, although phylogeographic analysis suggested potential international introductions, definitive source attribution and specific introduction routes could not be determined because of uneven global sampling, the availability of reference genomes in EnteroBase, and limited epidemiologic metadata. In addition, source-specific contextual information for some wastewater and poultry isolates was limited, few wastewater isolates were available, and import-lineage information was unavailable for the poultry isolates, limiting further epidemiologic interpretation. Third, long-read sequencing was performed only for selected representative isolates; consequently, plasmid structural diversity and transferability across the full collection could not be comprehensively assessed. Future studies comparing pESI-like plasmids across diverse *Salmonella* Infantis clades will be essential to clarify plasmid evolution, structural variation, and their roles in AMR, virulence, and host adaptation. Continued genomic surveillance and strengthened biosecurity could help improve early detection and risk assessment for further dissemination of multidrug-resistant *Salmonella* Infantis.

In conclusion, our genomic analysis suggests that *bla*_CTX-M-65_–positive *Salmonella Infantis* was introduced into South Korea on multiple occasions since 2018 and that isolates from poultry, wastewater, and humans showed close phylogenetic relatedness. Those findings are consistent with the emergence and intersectoral circulation of closely related multidrug-resistant lineages in South Korea and highlight the value of continued genomic surveillance across sectors.

Appendix Additional information on multiple introductions and cross-sector transmission of *Salmonella* e*nterica* serovar Infantis carrying *bla*_CTX-M-65_ gene, South Korea, 2022–2024.
