# Supplementary material for: Multiple Introductions and Cross-Sector Transmission of Salmonella enterica Serovar Infantis Carrying blaCTX-M-65 Gene, South Korea, 2022–2024
Source: Emerg Infect Dis. 2026 Oct;32(10):1599–608. doi: 10.3201/eid3210.251758 (PMC13632841; doi:10.3201/eid3210.251758)
Supplement: Supplementary file 1 [file 25-1758-Techapp-s1.pdf]

Article DOI: https://doi.org/10.3201/eid3210.251758

EID cannot ensure accessibility for supplementary materials supplied by authors. Readers who have difficulty accessing supplementary content should contact the authors for assistance.

Multiple Introductions and Cross-Sector Transmission of *Salmonella enterica* Serovar Infantis Carrying *bla*$_{CTX-M-65}$ Gene, South Korea, 2022–2024

Appendix

Appendix Table 1. Information of the *Salmonella* Infantis isolates sequenced in this study (n = 65).

ID	Country	Isolation year	Source	Reference	BioSample no.	Province
20221123	South_Korea	2022	Human	KDCA	SAMN51231600	Gangwon
20221564	South_Korea	2022	Human	KDCA	SAMN51231601	Gwangju
20221877	South_Korea	2022	Human	KDCA	SAMN51231602	Gyeongbuk
20221916	South_Korea	2022	Human	KDCA	SAMN51231603	Jeonnam
2023-205	South_Korea	2023	Human	KDCA	SAMN51231604	Gyeongbuk
2023-3078	South_Korea	2023	Human	KDCA	SAMN51231605	Incheon
2023-3262	South_Korea	2023	Human	KDCA	SAMN51231606	Incheon
2023-45	South_Korea	2023	Human	KDCA	SAMN51231607	Jeonbuk
2023-47	South_Korea	2023	Human	KDCA	SAMN51231608	Jeonbuk
2023-813	South_Korea	2023	Human	KDCA	SAMN51231609	Incheon
2023-816	South_Korea	2023	Human	KDCA	SAMN51231610	Incheon
2023-848	South_Korea	2023	Human	KDCA	SAMN51231611	Jeonnam
2024AST126	South_Korea	2024	Human	KDCA	SAMN51231612	Gyeongnam
23SAL-1035	South_Korea	2023	Human	KDCA	SAMN51231613	Gwangju
23SAL-120	South_Korea	2023	Human	KDCA	SAMN51231614	Daegu
23SAL-141	South_Korea	2023	Human	KDCA	SAMN51231615	Gwangju
23SAL-275	South_Korea	2023	Human	KDCA	SAMN51231616	Incheon
23SAL-3059	South_Korea	2023	Human	KDCA	SAMN51231617	Incheon
23SAL-3252	South_Korea	2023	Human	KDCA	SAMN51231618	Incheon
23SAL-3264	South_Korea	2023	Human	KDCA	SAMN51231619	Incheon
23SAL-3269	South_Korea	2023	Human	KDCA	SAMN51231620	Incheon
23SAL-707	South_Korea	2023	Human	KDCA	SAMN51231621	Gwangju
AN12	South_Korea	2023	Poultry	KDCA	SAMN51231622	Chungbuk
AN13	South_Korea	2023	Poultry	KDCA	SAMN51231623	Gyeonggi
AN15	South_Korea	2023	Poultry	KDCA	SAMN51231624	Incheon
AN17	South_Korea	2023	Poultry	KDCA	SAMN51231625	Gyeonggi
AN18	South_Korea	2023	Poultry	KDCA	SAMN51231626	Gangwon
AN21	South_Korea	2023	Poultry	KDCA	SAMN51231627	Gyeonggi
AN24	South_Korea	2023	Poultry	KDCA	SAMN51231628	Gyeonggi
AN29	South_Korea	2023	Poultry	KDCA	SAMN51231629	Gyeonggi
AN30	South_Korea	2023	Poultry	KDCA	SAMN51231630	Gyeonggi
AN31	South_Korea	2023	Poultry	KDCA	SAMN51231631	Jeonbuk
AN35	South_Korea	2023	Poultry	KDCA	SAMN51231632	Gyeonggi
AN36	South_Korea	2023	Poultry	KDCA	SAMN51231633	Incheon
AN7	South_Korea	2023	Poultry	KDCA	SAMN51231634	Chungnam
IC-2023-A0077	South_Korea	2023	Human	KDCA	SAMN51231635	Incheon
IC-2023-A0093	South_Korea	2023	Human	KDCA	SAMN51231636	Incheon
IC-2023-A0100	South_Korea	2023	Human	KDCA	SAMN51231637	Incheon
IC-2023-A0107	South_Korea	2023	Human	KDCA	SAMN51231638	Incheon
IC23-06-410	South_Korea	2023	Human	KDCA	SAMN51231639	Incheon
IC23-09-601	South_Korea	2023	Human	KDCA	SAMN51231640	Incheon
KRS20240099	South_Korea	2024	Human	KDCA	SAMN51231641	Sejong
KRS20240129	South_Korea	2024	Human	KDCA	SAMN51231642	Jeonnam

ID	Country	Isolation year	Source	Reference	BioSample no.	Province
KRS20240150	South_Korea	2024	Human	KDCA	SAMN51231643	Gwangju
KRS20240220	South_Korea	2024	Human	KDCA	SAMN51231644	Incheon
KRS20240311	South_Korea	2024	Human	KDCA	SAMN51231645	Incheon
KRS20240710	South_Korea	2024	Human	KDCA	SAMN51231646	Daejeon
KRS20240724	South_Korea	2024	Human	KDCA	SAMN51231647	Jeonnam
KRS20240725	South_Korea	2024	Human	KDCA	SAMN51231648	Jeonnam
KRS20240771	South_Korea	2024	Human	KDCA	SAMN51231649	Gwangju
KRS20240788	South_Korea	2024	Human	KDCA	SAMN51231650	Gwangju
KRS20240875	South_Korea	2024	Human	KDCA	SAMN51231651	Seoul
KRS20240876	South_Korea	2024	Human	KDCA	SAMN51231652	Seoul
KRS20241624	South_Korea	2024	Human	KDCA	SAMN51231653	Gangwon
KRS20241926	South_Korea	2024	Human	KDCA	SAMN51231654	Gyeongnam
KRS20242028	South_Korea	2024	Human	KDCA	SAMN51231655	Daegu
KRS20242121	South_Korea	2024	Human	KDCA	SAMN51231656	Gwangju
SAL-AN003	South_Korea	2023	Poultry	KDCA	SAMN51231657	Chungnam
SAL-AN005	South_Korea	2023	Poultry	KDCA	SAMN51231658	Chungbuk
SAL-AN014	South_Korea	2023	Poultry	KDCA	SAMN51231659	Gyeonggi
SAL-AN019	South_Korea	2023	Poultry	KDCA	SAMN51231660	Gangwon
SAL-WW035	South_Korea	2023	Wastewater	KDCA	SAMN51231661	Incheon
SAL-WW102	South_Korea	2023	Wastewater	KDCA	SAMN51231662	Incheon
SAL-WW105	South_Korea	2023	Wastewater	KDCA	SAMN51231663	Incheon
WW94	South_Korea	2023	Wastewater	KDCA	SAMN51231664	Incheon

Appendix Table 2. Information of the Korean *Salmonella* Infantis sequences used for the phylogenetic analysis in this study (n = 37).

ID	Country	Isolation year	Source	Reference	BioSample no.
230306_2	South_Korea	2023	Poultry	KU	SAMN48634499
230306_3	South_Korea	2023	Poultry	KU	SAMN48634500
230306_6	South_Korea	2023	Poultry	KU	SAMN48634501
230306_7	South_Korea	2023	Poultry	KU	SAMN48634502
230313_5	South_Korea	2023	Poultry	KU	SAMN48634503
230313_7	South_Korea	2023	Poultry	KU	SAMN48634504
230517_9	South_Korea	2023	Poultry	KU	SAMN48634505
230523_8	South_Korea	2023	Poultry	KU	SAMN48634506
230531_1	South_Korea	2023	Poultry	KU	SAMN48634507
230531_10	South_Korea	2023	Poultry	KU	SAMN48634511
230531_5	South_Korea	2023	Poultry	KU	SAMN48634508
230531_6	South_Korea	2023	Poultry	KU	SAMN48634509
230531_9	South_Korea	2023	Poultry	KU	SAMN48634510
230605_2	South_Korea	2023	Poultry	KU	SAMN48634512
230605_6	South_Korea	2023	Poultry	KU	SAMN48634513
230605_9	South_Korea	2023	Poultry	KU	SAMN48634514
230619_1	South_Korea	2023	Poultry	KU	SAMN48634515
230619_3	South_Korea	2023	Poultry	KU	SAMN48634516
230619_4	South_Korea	2023	Poultry	KU	SAMN48634517
230619_7	South_Korea	2023	Poultry	KU	SAMN48634518
230717_7	South_Korea	2023	Poultry	KU	SAMN48634519
IJCS5-22	South_Korea	2023	Poultry	NCBI	SAMN41563898
Z1322PSL0048	South_Korea	2023	Dog	NCBI	SAMN40424055
Z1322PSL0055	South_Korea	2023	Dog	NCBI	SAMN40424057
Z1323CSL0001	South_Korea	2023	Poultry	NCBI	SAMN40424058
Z1323CSL0002	South_Korea	2023	Poultry	NCBI	SAMN40424059
Z1323CSL0006	South_Korea	2023	Poultry	NCBI	SAMN40424060
Z1323CSL0014	South_Korea	2023	Poultry	NCBI	SAMN40424061
Z1323CSL0015	South_Korea	2023	Poultry	NCBI	SAMN40424062
Z1323CSL0016	South_Korea	2023	Poultry	NCBI	SAMN40424063
Z1323CSL0027	South_Korea	2023	Poultry	NCBI	SAMN40424065
Z1323CSL0034	South_Korea	2023	Poultry	NCBI	SAMN40424066
Z1323CSL0042	South_Korea	2023	Poultry	NCBI	SAMN40424067
Z1323HSL0002	South_Korea	2023	Human	NCBI	SAMN40424068
Z1323HSL0028	South_Korea	2023	Human	NCBI	SAMN40424070
Z1323HSL0030	South_Korea	2023	Human	NCBI	SAMN40424071
Z1323HSL0039	South_Korea	2023	Human	NCBI	SAMN40424072

Appendix Table 3. Information of the *Salmonella* Infantis sequences used for the phylogeographic analysis in this study (n = 79).

ID	Source Niche	Collection Year	Country	Accession
SAL_VB0145AA_AS	Human	2019	United Kingdom	SRR10420542
SAL_BC4614AA_AS	Human	2019	United Kingdom	SRR11142769
SAL_LB5040AA_AS	Human	2019	United Kingdom	SRR8936136
SAL_SB1832AA_AS	Human	2019	United Kingdom	SRR10236706
SAL_MC5286AA_AS	Human	2020	United Kingdom	SRR13332540
SAL_KD9552AA_AS	Human	2023	United Kingdom	SRR29528188
SAL_WA6094AA_AS	Human	2017	United Kingdom	SRR6233871
SAL_QA0307AA_AS	Human	2016	United Kingdom	SRR5193958
SAL_WA3817AA_AS	Human	2017	United Kingdom	SRR6191367
SAL_PC9116AA_AS	Human	2013	United Kingdom	SRR14774768
SAL_KB9443AA_AS	Human	2019	United Kingdom	SRR8774341
SAL_KD0155AA_AS	Human	2023	United Kingdom	SRR29327283
SAL_PD9891AA_AS	Human	2024	United Kingdom	SRR30881853
SAL_HD3790AA_AS	Human	2024	United Kingdom	SRR28414858
SAL_GD8811AA_AS	Human	2023	United Kingdom	SRR28115553
SAL_KC7222AA_AS	Human	2020	United Kingdom	SRR12770068
SAL_ID8185AA_AS	Human	2024	United Kingdom	SRR29047444
SAL_JD4371AA_AS	Human	2023	United Kingdom	SRR29209795
SAL_MB0334AA_AS	Human	2019	United Kingdom	SRR9054491
SAL_FB4761AA_AS	Human	2018	United Kingdom	SRR8131534
SAL_LB2306AA_AS	Human	2019	United Kingdom	SRR8874191
SAL_AB9177AA_AS	Human	2018	United Kingdom	SRR7184567
SAL_PC8038AA_AS	Human	2021	United Kingdom	SRR14724715
SAL_PC5935AA_AS	Human	2021	United Kingdom	SRR14616327
SAL_KD1823AA_AS	Human	2023	United Kingdom	SRR29367158
SAL_JD1059AA_AS	Human	2024	United Kingdom	SRR29091979
SAL_IB6668AA_AS	Human	2018	United Kingdom	SRR8499050
SAL_BB8154AA_AS	Human	2018	United Kingdom	SRR7401969
SAL_HD5351AA_AS	Poultry	2022	Saudi Arabia	SRR28556680
SAL_WC7006AA_AS	Poultry	2020	Saudi Arabia	SRR21549212
SAL_WC7005AA_AS	Poultry	2020	Saudi Arabia	SRR21549211
SAL_WC7004AA_AS	Poultry	2020	Saudi Arabia	SRR21549210
SAL_WC7013AA_AS	Poultry	2020	Saudi Arabia	SRR21549219
SAL_WC7014AA_AS	Poultry	2020	Saudi Arabia	SRR21549220
SAL_WC7012AA_AS	Poultry	2020	Saudi Arabia	SRR21549218
SAL_WC7003AA_AS	Poultry	2020	Saudi Arabia	SRR21549209
SAL_WC7002AA_AS	Poultry	2020	Saudi Arabia	SRR21549208
SAL_WC7009AA_AS	Poultry	2020	Saudi Arabia	SRR21549215
SAL_WC7008AA_AS	Poultry	2020	Saudi Arabia	SRR21549214
SAL_WC7007AA_AS	Poultry	2020	Saudi Arabia	SRR21549213
SAL_CB1551AA_AS	Human	2013	Peru	SRR7450530
SAL_MA5436AA_AS	Human	2012	Peru	SRR3931722
SAL_MA5435AA_AS	Human	2012	Peru	SRR3931721
SAL_CB1552AA_AS	Human	2013	Peru	SRR7450529
SAL_CB1513AA_AS	Human	2013	Peru	SRR7450568
SAL_CB1534AA_AS	Human	2014	Peru	SRR7450547
SAL_MA5483AA_AS	Human	2012	Peru	SRR3931744
SAL_MA5465AA_AS	Human	2014	Peru	SRR3931698
SAL_CB1537AA_AS	Human	2014	Peru	SRR7450544
SAL_CB1556AA_AS	Human	2012	Peru	SRR7450525
SAL_MA5414AA_AS	Human	2014	Peru	SRR3931694
SAL_CB1500AA_AS	Human	2014	Peru	SRR7450581
SAL_CB1833AA_AS	Human	2011	Peru	SRR7456275
SAL_MA5482AA_AS	Human	2014	Peru	SRR3931701
SAL_CB1865AA_AS	Human	2012	Peru	SRR7456243
SAL_CB1872AA_AS	Human	2012	Peru	SRR7456236
SAL_MA5474AA_AS	Human	2012	Peru	SRR3931761
SAL_MA5473AA_AS	Human	2012	Peru	SRR3931755
SAL_MA5420AA_AS	Human	2014	Peru	SRR3931702
SAL_MA5469AA_AS	Human	2012	Peru	SRR3931716
SAL_CB1541AA_AS	Human	2013	Peru	SRR7450540
SAL_MA5434AA_AS	Human	2012	Peru	SRR3931720
SAL_CB1502AA_AS	Human	2013	Peru	SRR7450579
SAL_CB1859AA_AS	Human	2012	Peru	SRR7456249
SAL_MA5429AA_AS	Human	2012	Peru	SRR3931715
SAL_GB0395AA_AS	Human	2012	Peru	SRR7450536
SAL_MA5472AA_AS	Human	2012	Peru	SRR3931749
SAL_CB1501AA_AS	Human	2013	Peru	SRR7450580

ID	Source Niche	Collection Year	Country	Accession
SAL_PC8216AA_AS	Human	2019	Germany	ERR4832874
SAL_PC8230AA_AS	Human	2019	Germany	ERR4832888
SAL_PC8238AA_AS	Human	2019	Germany	ERR4832896
SAL_LC5270AA_AS	Human	2017	Canada	SRR13019251
SAL_MC3087AA_AS	Human	2017	Canada	SRR13243602
SAL_PC6706AA_AS	Human	2015	Canada	SRR14660121
SAL_RC8303AA_AS	Human	2017	Canada	SRR15242780
SAL_OC7666AA_AS	Human	2018	Canada	SRR14215703
SAL_DD9628AA_AS	Human	2018	Australia	SRR26388918
SAL_ID0268AA_AS	Human	2023	Australia	SRR28855333
SAL_ID1369AA_AS	Human	2023	Australia	SRR28854730

Appendix Table 4. Minimum inhibitory concentrations (µg/mL) of the *Salmonella* Infantis isolates tested in this study (n = 65).

	Minimum inhibitory concentrations (µg/mL)														
Isolates	CIP	IMI	COL	CTX	AMP	AXO	TET	CHL	GEN	AZI	AMI	NAL	SXT	FOX	CAZ
23SAL-120	0.25	<1	<2	>32	>64	>32	128	>32	4	8	<4	>128	<1/19	16	>16
20221877	<0.03	<1	<2	32	>64	>32	128	>32	<1	4	<4	>128	>16/304	<4	4
2023-205	0.5	<1	<2	32	>64	>32	128	>32	8	8	<4	>128	<1/19	8	2
20221564	0.25	<1	<2	32	>64	>32	128	>32	16	8	<4	>128	>16/304	8	2
23SAL-141	0.12	<1	<2	>32	>64	>32	64	>32	16	<2	<4	128	>16/304	<4	2
23SAL-707	0.5	8	<2	>32	>64	>32	>128	>32	8	8	<4	>128	>16/304	32	16
23SAL-1035	0.25	<1	<2	32	>64	>32	128	>32	4	4	<4	>128	<1/19	16	2
20221123	0.5	<1	<2	>32	>64	>32	128	>32	8	8	<4	>128	<1/19	16	8
2023-813	0.25	<1	<2	32	>64	32	128	>32	4	4	<4	128	>16/304	8	2
2023-816	0.12	<1	<2	32	>64	>32	128	>32	8	<2	<4	64	>16/304	<4	2
2023-3078	0.12	<1	<2	32	>64	>32	64	>32	8	<2	<4	64	>16/304	<4	2
IC-2023-A0077	0.5	<1	<2	>32	>64	>32	>128	>32	8	8	<4	>128	>16/304	16	8
IC-2023-A0093	0.5	<1	<2	>32	>64	>32	>128	>32	16	8	<4	>128	>16/304	16	8
IC-2023-A0100	0.5	<1	<2	>32	>64	>32	>128	>32	8	8	<4	>128	>16/304	16	8
IC-2023-A0107	0.5	<1	<2	>32	>64	>32	>128	>32	8	8	<4	>128	>16/304	8	4
23SAL-3252	0.12	<1	4	32	>64	>32	64	>32	8	<2	<4	128	>16/304	<4	8
2023-3262	0.25	<1	<2	32	>64	>32	128	16	4	8	<4	>128	<1/19	16	2
23SAL-3264	0.5	<1	<2	>32	>64	>32	>128	16	8	8	<4	>128	<1/19	16	2
23SAL-3269	0.5	<1	<2	>32	>64	>32	>128	16	<1	<2	<4	>128	<1/19	16	>16
23SAL-3059	0.5	<1	<2	32	>64	>32	128	>32	8	8	<4	>128	>16/304	16	2
IC23-06-410	8	<1	<2	>32	>64	>32	>128	>32	8	8	<4	>128	>16/304	16	4
IC23-09-601	0.5	<1	<2	>32	>64	>32	>128	>32	8	8	<4	>128	>16/304	16	8
2023-45	0.5	<1	<2	>32	>64	>32	>128	>32	8	8	<4	>128	>16/304	16	4
2023-47	0.5	<1	<2	>32	>64	>32	>128	>32	8	8	<4	>128	>16/304	16	2
20221916	0.12	<1	<2	32	>64	>32	64	>32	16	<2	<4	64	>16/304	<4	2
2023-848	0.25	<1	<2	>32	>64	>32	128	>32	8	8	<4	>128	<1/19	16	>16
AN12	8	<1	<2	>32	>64	>32	128	>32	4	8	<4	>128	>16/304	>32	16
AN13	0.5	<1	<2	>32	>64	>32	128	>32	8	8	<4	>128	>16/304	16	2
SAL-AN014	0.5	<1	<2	32	>64	>32	>128	32	4	8	<4	>128	>16/304	16	4
AN15	0.5	<1	<2	>32	>64	>32	128	>32	16	8	<4	>128	>16/304	16	4
AN17	0.5	<1	<2	32	>64	>32	128	>32	16	8	<4	>128	>16/304	16	4
AN18	0.25	<1	<2	32	>64	>32	128	>32	16	8	<4	>128	>16/304	16	4
SAL-AN019	0.12	<1	<2	32	>64	>32	64	>32	8	8	<4	128	>16/304	16	2
AN21	0.5	<1	<2	>32	>64	>32	>128	>32	8	16	<4	>128	>16/304	32	4
AN24	0.12	<1	<2	32	>64	>32	128	>32	8	8	<4	128	>16/304	16	2
AN29	0.25	<1	<2	>32	>64	>32	128	>32	8	8	<4	128	>16/304	16	2
SAL-AN003	0.5	<1	<2	32	>64	>32	128	>32	8	8	<4	>128	>16/304	16	2
AN30	0.5	<1	<2	>32	>64	>32	>128	>32	8	8	<4	>128	>16/304	16	4
AN31	0.5	<1	<2	>32	>64	>32	>128	>32	8	8	<4	>128	>16/304	16	4
AN35	0.5	<1	<2	>32	>64	>32	>128	>32	16	8	<4	>128	>16/304	16	4
AN36	8	<1	<2	>32	>64	>32	>128	>32	32	8	<4	>128	>16/304	32	4
AN7	0.12	<1	<2	32	>64	>32	128	>32	8	4	<4	128	>16/304	16	2
SAL-AN005	0.25	<1	<2	32	>64	>32	128	>32	8	4	<4	128	>16/304	16	2
23SAL-275	0.5	<1	<2	>32	>64	>32	>128	32	8	16	<4	>128	<1/19	16	8
SAL-WW102	0.12	<1	<2	>32	>64	>32	128	>32	16	8	<4	>128	>16/304	16	8
SAL-WW105	0.5	<1	<2	32	>64	>32	128	>32	8	8	<4	>128	>16/304	16	2
SAL-WW035	0.5	<1	<2	32	>64	>32	128	>32	8	8	<4	>128	>16/304	16	4
WW94	0.25	<1	<2	32	>64	>32	128	>32	4	8	<4	>128	>16/304	16	2
2024AST126	0.5	<1	<2	>32	>64	>32	128	>32	8	8	<4	128	>16/304	16	4
KRS20240099	0.25	<1	<2	32	>64	32	64	>32	8	4	<4	128	<1/19	8	2
KRS20240129	0.25	<1	<2	32	>64	>32	128	>32	16	8	<4	128	<1/19	8	2

	Minimum inhibitory concentrations (µg/mL)														
Isolates	CIP	IMI	COL	CTX	AMP	AXO	TET	CHL	GEN	AZI	AMI	NAL	SXT	FOX	CAZ
KRS20240150	0.5	<1	<2	>32	>64	>32	128	16	8	8	<4	>128	>16/304	8	2
KRS20240220	0.25	<1	<2	>32	>64	>32	128	>32	16	8	<4	128	<1/19	8	2
KRS20240311	0.25	<1	<2	>32	>64	>32	128	>32	8	4	<4	128	>16/304	8	4
KRS20240710	0.5	<1	<2	>32	>64	>32	128	>32	32	8	<4	>128	>16/304	16	16
KRS20240724	0.25	<1	<2	32	>64	>32	128	>32	16	8	<4	128	>16/304	8	2
KRS20240725	0.25	<1	<2	16	>64	32	128	>32	4	8	<4	128	>16/304	8	4
KRS20240771	0.25	<1	<2	>32	>64	>32	128	>32	8	8	<4	>128	>16/304	8	4
KRS20240788	0.25	<1	<2	32	>64	>32	128	>32	16	8	8	128	>16/304	8	4
KRS20240875	0.5	<1	<2	>32	>64	>32	128	>32	<1	8	<4	<2	<1/19	<4	<1
KRS20240876	0.5	<1	<2	>32	>64	>32	>128	>32	32	8	<4	>128	>16/304	>32	>16
KRS20241624	0.25	<1	<2	>32	>64	>32	128	>32	4	4	<4	>128	>16/304	8	2
KRS20241926	0.12	<1	<2	32	>64	>32	64	>32	16	<2	<4	64	>16/304	<4	2
KRS20242028	0.25	<1	<2	>32	>64	>32	128	>32	8	4	<4	128	>16/304	8	2
KRS20242121	0.12	<1	<2	>32	>64	>32	64	>32	8	<2	<4	64	>16/304	<4	4
No. resistant isolates	3	1	1	65	65	65	65	61	53	0	0	64	52	5	7
(%)	4.62	1.54	1.54	100	100	100	100	93.85	81.54	0.00	0.00	98.46	80.00	7.69	10.77

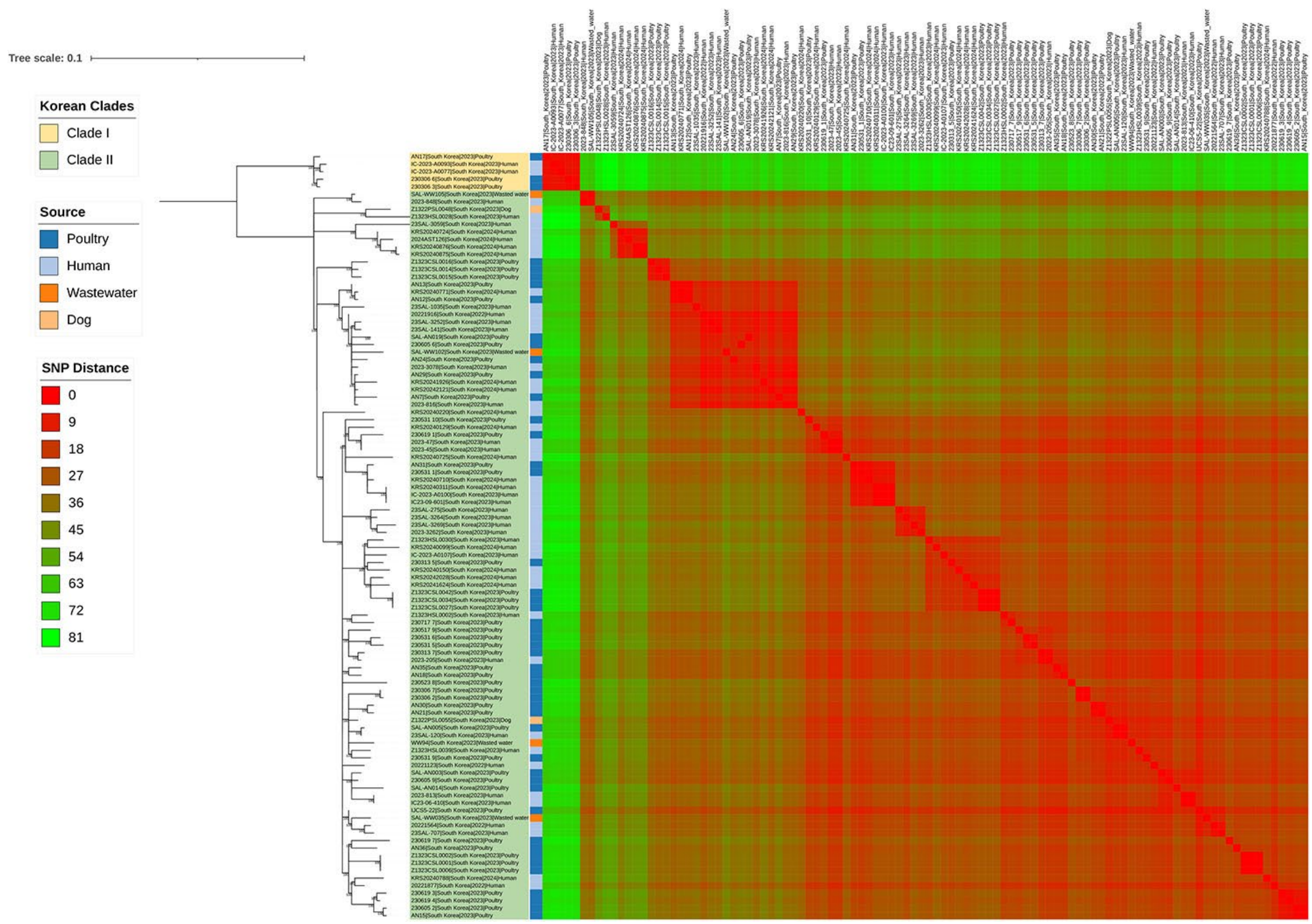

Appendix Figure 1. Maximum-likelihood phylogenetic tree of *bla*CTX-M-65-positive *Salmonella* Infantis isolates from South Korea (n = 102). An adjacent heatmap illustrates SNP distances among the isolates.

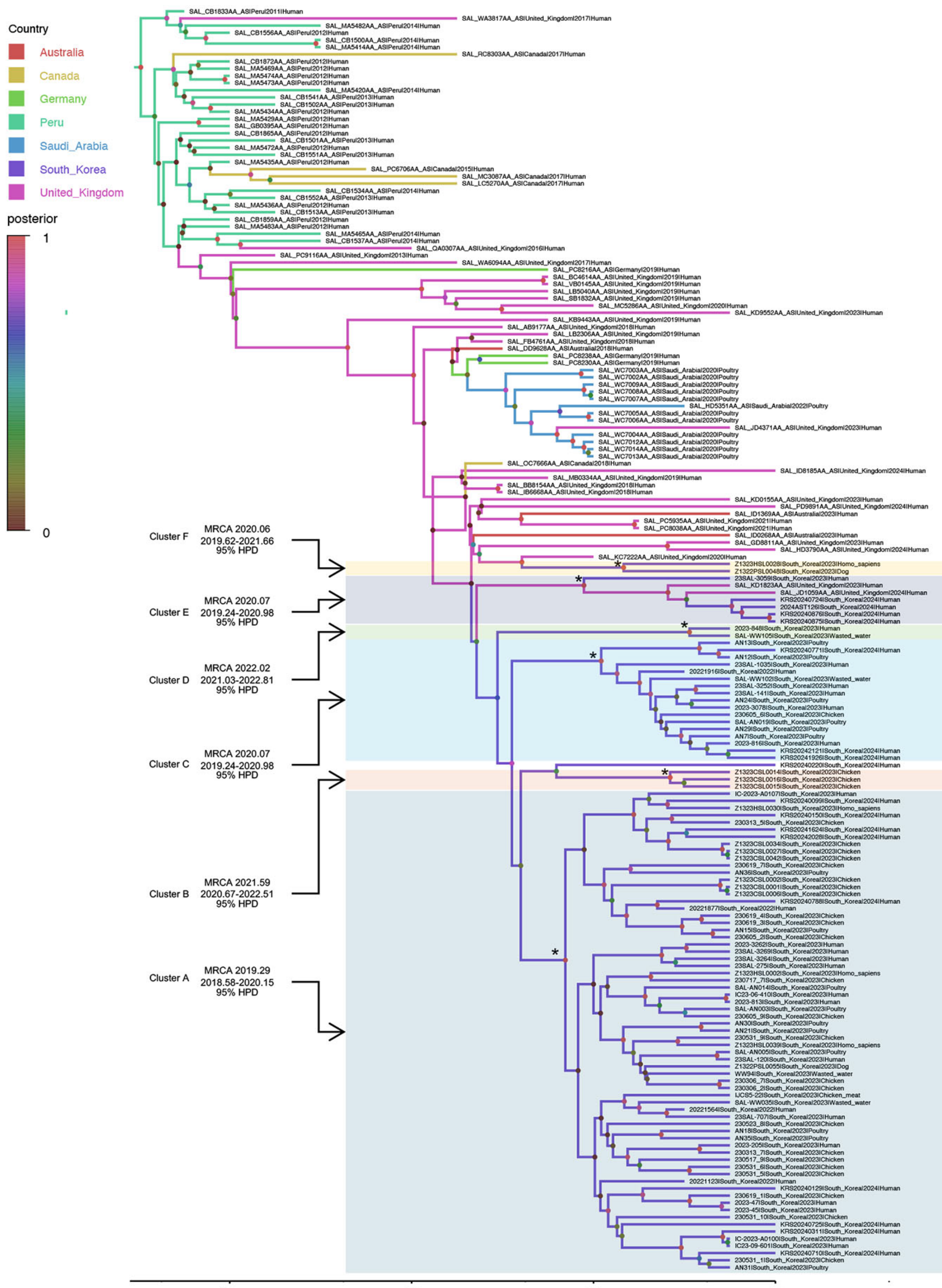

Appendix Figure 2. Full Bayesian phylogeny corresponding to the simplified tree shown in Figure 3.

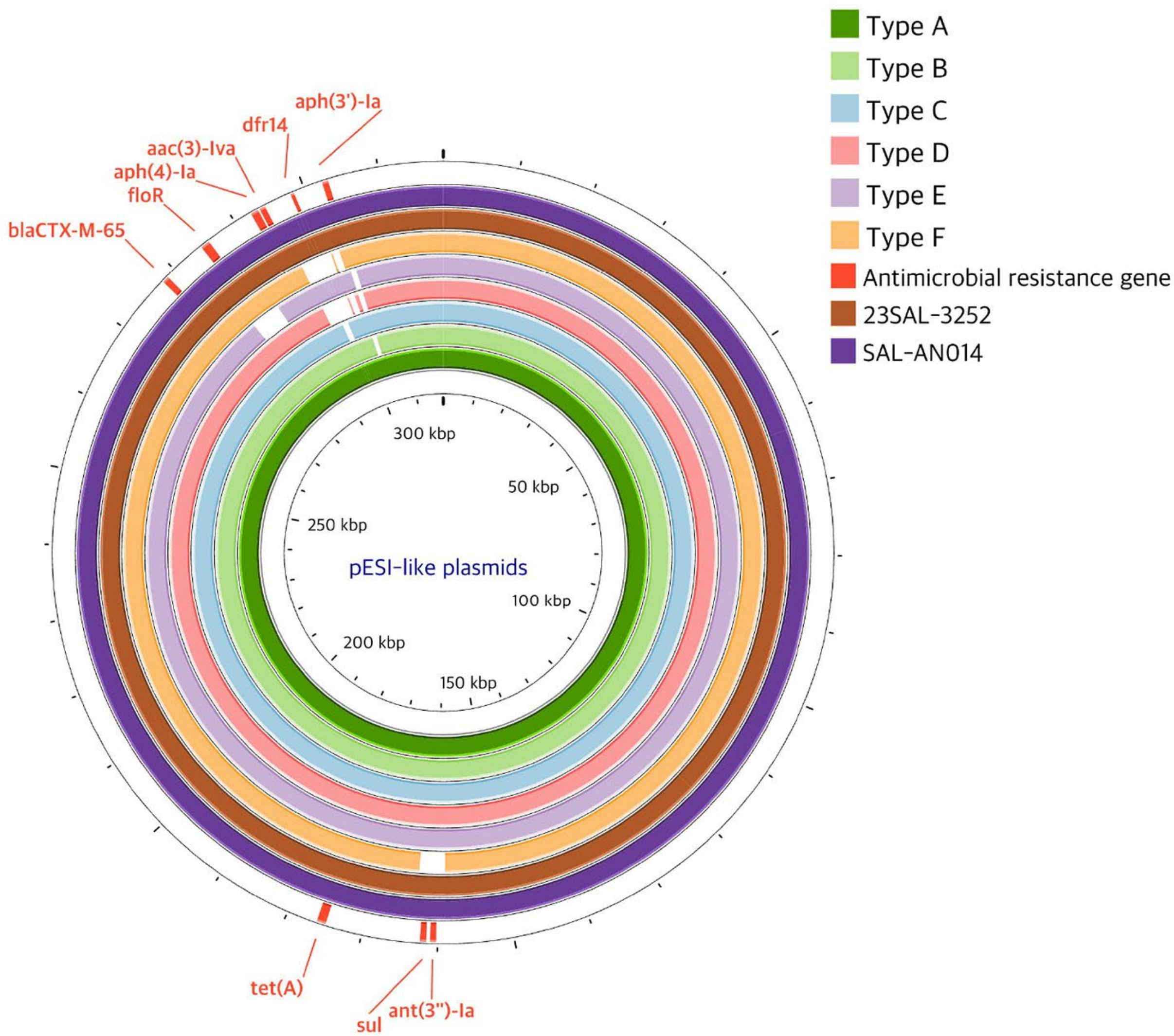

Appendix Figure 3. Circular genomic comparison of pESI-like plasmid variants identified in South Korean *Salmonella* Infantis, generated using Proksee. Colored rings represent plasmid types A–F together with 2 newly sequenced clade II plasmids (23SAL-3252 and SAL-AN014), and red bars indicate the positions of annotated antimicrobial resistance genes.
